# Molecular Pathogenesis of Connective Tissue Disease-Associated Pulmonary Arterial Hypertension: A Narrative Review

**DOI:** 10.3390/biom15060772

**Published:** 2025-05-27

**Authors:** Fu-Chiang Yeh, I-Ting Tsai, I-Tsu Chyuan

**Affiliations:** 1 Division of Rheumatology, Immunology and Allergy, Department of Internal Medicine, Tri-Service General Hospital, National Defense Medical Center, Taipei 114202, Taiwan; moses0722@gmail.com; 2 Department of Internal Medicine, Tri-Service General Hospital, National Defense Medical Center, Taipei 114202, Taiwan; doc96118@gmail.com; 3 School of Medicine, National Tsing Hua University, Hsinchu 30013, Taiwan; 4 Department of Internal Medicine, Cathay General Hospital, Taipei 10630, Taiwan; 5 Department of Medical Research, Cathay General Hospital, Taipei 10630, Taiwan

**Keywords:** pulmonary arterial hypertension, connective tissue diseases, pathogenesis, immune dysregulation, serum biomarkers, animal models

## Abstract

Pulmonary arterial hypertension (PAH) is a lethal condition marked by the proliferation and remodeling of small pulmonary arteries, ultimately leading to right ventricular hypertrophy and right heart failure. PAH secondary to connective tissue diseases (CTDs) is a progressive complication with a complex pathogenesis that results in the reduced efficacy of vasodilation-based therapies and poor clinical outcomes. Systemic sclerosis is the most commonly associated CTD with PAH in Western countries and has been most extensively investigated. Systemic lupus erythematosus and other CTDs may also be associated with PAH; however, they are less studied. In this review, we explore the general pathobiology of PAH, with a particular emphasis on recent advances in the molecular pathogenesis of CTD-PAH, including endothelial cell dysfunction, dysregulated cell proliferation and vascular remodeling, extracellular matrix remodeling, in situ thrombosis, right ventricular dysfunction, genetic aberrations, and immune dysregulation. We also conduct a thorough investigation into the potential serum biomarkers and immune dysregulation associated with CTD-PAH, summarizing the associated autoantibodies, cytokines, and chemokines. Furthermore, relevant animal models that may help unravel the pathogenesis and contribute to the development of new treatments are also reviewed.

## 1. Introduction

Pulmonary arterial hypertension (PAH) is a life-threatening condition characterized by the proliferation and remodeling of small pulmonary arteries, resulting in progressive pulmonary vascular occlusion and increased pulmonary vascular resistance (PVR), which ultimately leads to right ventricular hypertrophy (RVH) and right heart failure (HF). The disease is diagnosed through right heart catheterization (RHC), with hemodynamic criteria indicating precapillary pulmonary hypertension (PH), including mean pulmonary arterial pressure (mPAP) > 20 mmHg, PVR > 2 Woods Units, and pulmonary arterial wedge pressure (PAWP) ≤15 mmHg. PAH can be idiopathic (IPAH), inherited, drug- or toxin-induced, or secondary to an underlying disease [1].

Connective tissue diseases (CTDs), a group of diseases caused by autoimmunity and chronic inflammation, are the most common conditions linked to PAH, with CTD-PAH being the second leading cause of PAH after the idiopathic form [2]. Among all CTDs, systemic sclerosis (SSc) is the most associated with PAH, affecting 8–12% of SSc patients and accounting for nearly 75% of CTD-PAH cases in Western countries. PAH is a major cause of death in SSc and carries a worse prognosis than IPAH [3]. Additionally, PAH occurs in 1–5% of systemic lupus erythematosus (SLE) patients [4], with SLE being the most common underlying condition in CTD-PAH in Japan, Korea, China, and Taiwan, affecting 29–57% of CTD-PAH patients [5]. PAH has also rarely been reported in primary Sjögren’s disease (pSS), mixed connective tissue disease (MCTD), idiopathic inflammatory myopathies (IIM), and rheumatoid arthritis (RA) [6].

Diagnosing PAH in patients with CTDs can be challenging due to non-specific symptoms that may overlap with those of underlying CTDs and a lack of specific diagnostic markers. As a result, CTD-PAH is frequently diagnosed at a late stage, when pulmonary vascular damage has become irreversible. Furthermore, current vasodilation-based therapies for PAH tend to be less effective in CTD-PAH, with CTD-PAH patients generally experiencing worse outcomes compared to those with IPAH in most trials [7,8]. To improve early diagnosis, the DETECT and ASIG algorithms were developed as multi-modality screening tools for PAH in SSc patients [9,10]. Recently, Taiwanese experts also proposed a new screening protocol for non-SSc CTD-PAH [5].

Recent advancements have greatly enhanced our understanding of the pathogenesis of PAH, with a particular emphasis on IPAH. The proposed mechanisms include endothelial cell (EC) dysfunction, dysregulated cell proliferation and vascular remodeling, extracellular matrix (ECM) remodeling, in situ thrombosis, right ventricular (RV) dysfunction, genetic aberrations, and immune dysregulation. In this review, we discuss the general pathobiology of PAH while focusing on updating the molecular pathogenesis of CTD-PAH, starting with SSc-PAH followed by non-SSc CTD-PAH. We also offer an in-depth exploration of immune dysregulation in CTD-PAH, summarizing key autoantibodies, cytokines, chemokines, and potential serum biomarkers. Furthermore, we highlight relevant animal models that could help unravel the pathogenesis of CTD-PAH and contribute to the development of new treatments.

## 2. Systemic Sclerosis-Associated PAH (SSc-PAH)

SSc is a complex disease involving autoimmunity, vascular damage, and fibrosis that affects both the skin and internal organs. A major complication of SSc is PAH, a leading cause of death in these patients [11]. The development of PAH is driven by a combination of several factors, including the dysfunction of various vascular cells, inflammation, and intricate intracellular signaling pathways, contributing to vascular damage and remodeling. SSc-PAH is the most common in CTD-PAH in Western countries and has the worst prognosis [12,13,14].

### 2.1. Pathology

Pulmonary vascular remodeling in SSc-PAH is typically characterized by intimal hyperplasia, medial hypertrophy, and adventitial thickening accompanied by inflammatory infiltrates. Both arterial and venous remodeling were observed in SSc-PAH, with common pathological features of intraluminal thrombosis and intimal fibrosis. Compared to IPAH patients, SSc-PAH patients demonstrated more obstructive venopathy, with the fibrosis of venules or veins with capillary congestion, like in pulmonary veno-occlusive disease, but less plexogenic arteriopathy [15,16,17].

### 2.2. Pathobiology

PAH is driven by several common pathophysiological mechanisms, including EC dysfunction, dysregulated cell proliferation and vascular remodeling, intracellular signaling, in situ thrombosis, ECM remodeling, and immune dysregulation. Furthermore, the condition leads to RVH as a compensatory response to the elevated PVR, which aims to maintain pulmonary blood flow. However, this adaptation may ultimately progress to right HF. Due to the limited number of studies on SSc-PAH, here, we review the general pathobiology of PAH and emphasize the findings relevant to SSc-PAH.

#### 2.2.1. Endothelial Cell Dysfunction

The pulmonary endothelium produces various vasoactive mediators, including prostacyclin (PGI_2_), nitric oxide (NO), endothelin 1 (ET-1), and 5-hydroxytryptamine (5-HT), and can mediate inflammation and regulate platelet function and vascular tone. The disruption of EC homeostasis is the main cause of PAH [18]. In PAH, an imbalance between vasodilatory and vasoconstrictive mediators occurs within the lung tissue and circulation, leading to excessive pulmonary vasoconstriction. Additionally, emerging evidence supports the involvement of reactive oxygen species (ROS) overproduction and endothelial-to-mesenchymal transition (EndMT) in pulmonary endothelial dysfunction and the pathogenesis of SSc-PAH.

- Prostacyclin

PGI_2_ synthase, located on the pulmonary ECs, produces PGI_2_ from PGH_2_. PGI_2_ is a potent vasodilator that induces smooth muscle relaxation and suppresses platelet aggregation by increasing cyclic adenosine monophosphate levels [19]. In contrast, thromboxane A_2_ (TXA_2_), produced by thromboxane synthase in activated platelets, promotes vasoconstriction and platelet aggregation. The imbalance between PGI_2_ and TXA_2_, with a reduction in PGI_2_ release and an increase in TXA_2_ production, was noted in PAH. This imbalance contributed to proliferation, vasoconstriction, and thrombosis [19,20,21]. In addition, the first targeted therapy for PAH, which involved replacing prostacyclin with intravenous epoprostenol, has been available since 1995.

- Nitric oxide

Within the pulmonary circulation, NO, generated by endothelial NO synthase (eNOS) from L-arginine, plays a central role in endothelium-dependent vasodilation [22,23]. It promotes the production of cyclic guanosine monophosphate (cGMP) from guanosine triphosphate by activating soluble guanylyl cyclase. cGMP has a strong vasodilatory effect and helps regulate pulmonary vascular structure by inhibiting smooth muscle cell (SMC) proliferation. Furthermore, cGMP prevents thrombosis and platelet aggregation, thereby contributing to the maintenance of pulmonary vascular homeostasis. The levels of intracellular cGMP are regulated by phosphodiesterase type 5 [24].

Dysfunction of the NO signaling pathway has been implicated in the development of PAH. For instance, lower levels of NO and the reduced expression of eNOS in the pulmonary endothelium have been noted in PAH patients and correlate with the severity of histological changes [25]. Gene transfer of *eNOS* into the lungs of *eNOS* knockout (*eNOS^-/-^*) mice resulted in increased eNOS expression, enhanced activity, and elevated cGMP levels, which partially mitigated pulmonary arterial pressure (PAP) and PVR [26]. Due to its crucial role in regulating pulmonary vascular tone and remodeling, exhaled NO has been studied as a biomarker for PAH and SSc-PAH [27,28].

- Endothelin 1

ET-1 is a potent vasoconstrictor and a mild mitogen for SMCs, playing a key role in regulating vascular tone through its interaction with endothelin receptor type A (ET_A_R) and endothelin receptor type B (ET_B_R) on pulmonary artery smooth muscle cells (PASMCs). ET-1 is found in plexiform lesions and is overexpressed in both the circulation and the lungs of patients with PAH [29,30].

The activation of ET_A_R promotes vasoconstriction and participates in the pathogenesis of PAH, while ET_B_R activation has a more modest effect, inducing vessel wall relaxation by stimulating the release of NO and prostaglandins [31]. Serum levels of anti-ET_A_R antibodies were elevated in both SSc patients with and without PAH compared to other groups of PH. The predictive values of anti-ET_A_R antibodies for PAH development among SSc patients and prognosis in SSc-PAH patients were demonstrated [32,33].

In addition, ET-1 promotes fibrogenesis by interacting with matrix metalloproteinases (MMPs), driving connective tissue remodeling [34]. It plays an important role in mediating inflammation, proliferation, and fibrosis. Elevated ET-1 expression was detected in the fibroblasts and ECs of SSc patients, correlating with the severity of fibrotic phenotype and the number of scars [35]. Furthermore, higher ET-1 serum levels were noted in SSc-PAH patients [36,37].

- 5-Hydroxytryptamine

5-HT, commonly known as serotonin, is synthesized in the ECs lining the pulmonary artery. It can induce vascular constriction and remodeling by affecting the SMCs and fibroblasts beneath the vessel walls. This compound is also a strong vasoconstrictor and is upregulated in patients with PAH [38].

- Reactive oxygen species

In SSc patients, circulating pro-oxidant factors are associated with intimal hyperplasia and vascular dysfunction [39]. Furthermore, increased levels of intracellular ROS in vascular smooth muscle cells (VSMCs) were detected in SSc-PAH patients compared to healthy controls (HCs) and SSc patients without PAH (SSc-nonPAH). NADPH-derived ROS production from SSc-PAH sera was found to activate collagen synthesis in VSMCs [40].

- Endothelial-to-mesenchymal transition

Some evidence suggests that EndMT plays a role in the development of PAH. In this process, ECs adopt a mesenchymal phenotype, typically seen in SMCs, which enables them to migrate and remodel the ECM. The cells with both mesenchymal and EC markers, along with the identification of ribonucleic acid (RNA) and protein signatures linked to EndMT, were found in the intimal lesions of pulmonary arteries from patients with severe PAH. This suggests that the process of EndMT may be a key mechanism in neointima formation. Additionally, molecular factors driving this pathological transition, such as vimentin phosphorylation and twist overexpression, have been identified in both human PAH and animal models of PH [41,42]. Several factors have been implicated in triggering EndMT in PAH, including high pulsatile flow, high shear stress, and the imbalance of bone morphogenetic proteins (BMPs)/the transforming growth factor (TGF)-β signaling axis [43,44].

The BMPs and TGF-βs belong to the TGF-β superfamily, which modulates the processes of tissue homeostasis, including cell differentiation, proliferation, adhesion, and migration. Hopper et al. showed that reduced bone morphogenetic protein receptor type 2 (BMPR2) signaling in pulmonary ECs resulted in the increased expression of High Mobility Group AT-Hook 1, which may facilitate the binding of pro-EndMT transcription factors [42]. The dysfunction of BMPR2 signaling is found in PAH patients, and BMPR2 mutations have been identified in some familial PAH (FPAH) patients [45]. Similarly, the dysregulated expression of TGF-β isoforms was found in SSc-PAH patients, suggesting that the TGF-β pathway may be involved in the pathogenesis of SSc-PAH [46].

#### 2.2.2. Dysregulated Cell Proliferation and Vascular Remodeling

- Pulmonary endothelium

In PAH, the dysfunctional pulmonary endothelium contributes to both the initiation and progression of vascular remodeling, affecting the structure and function of the blood vessels. For instance, the endothelium of distal pulmonary arteries adopts a pro-inflammatory phenotype, marked by an overproduction of key cytokines, chemokines, and growth factors, which contributes to the proliferation and survival of surrounding pulmonary vascular cells [47,48,49]. In addition, pulmonary vascular cells from patients with IPAH, including ECs, fibroblasts, pericytes, and PASMCs, demonstrate an enhanced growth response to growth factors, altered metabolic processes, and greater resistance to apoptosis compared to control cells [50,51,52,53,54,55,56,57,58].

Some main processes involving the ECs, including EC injury, defective vasculogenesis, defective angiogenesis, and EndMT, play an important role in the pathogenesis of both SSc and SSc-PAH. In the pulmonary vasculature of SSc-PAH patients, endothelial dysfunction contributes to the aggregation and expansion of α-smooth muscle actin cells and collagen type-1 positive cells, while EndMT leads to inflammatory infiltration and vascular remodeling [59].

- Pericytes

Pericytes are important in maintaining vascular integrity and stabilization. They also play a crucial role in regulating vascular tone and the proliferation of ECs. Disruption in endothelial–pericyte interactions can lead to pathological vascular remodeling [54,60]. The marked increase in pericyte numbers within the peripheral pulmonary arteries of PAH patients indicates that pericytes may be involved in the pathogenesis of PAH. In addition, pulmonary EC-derived fibroblast growth factor-2 and interleukin (IL)-6 can induce pericyte migration and proliferation, and upregulated TGF-β has been implicated in promoting the differentiation of pericytes into contractile smooth muscle-like cells in PAH [54].

- Smooth muscle cells

Generally, the hyperplasia of SMCs and the gradual obliteration of pulmonary vessels are caused by inherent intrinsic abnormalities in the resident vascular cells, along with disrupted signaling within the cellular microenvironment of the pulmonary artery walls. SMCs can regulate blood vessel tone by changing morphology, migration, and proliferation rates. A proliferative and synthetic phenotype occurs when vascular injury happens and can be induced by platelet-derived growth factors. This phenotype leads to intimal hyperplasia and may induce the development of several vascular diseases [61]. The proliferation and migration of VSMCs were found to mediate PAH development in PAH model rats, activated by IL-17 [62].

Furthermore, PASMCs from IPAH patients exhibit significantly greater proliferative capacity in vitro compared to controls [63]. The hyper-proliferative vascular cells in PAH show altered metabolic pathways, leading to increased aerobic glycolysis, a phenomenon also seen in cancer cells and referred to as the Warburg effect. Notably, the significant upregulation of glucose transporter 1 is observed in both proliferating ECs and SMCs in patients and animal models [64].

- Fibroblasts

Fibroblasts are the source of ECM and the progenitors for some mesenchymal cells during tissue remodeling. The migration of myofibroblasts into the vascular adventitia leading to vessel thickening and vascular fibrosis is a key factor of vascular remodeling in PAH and SSc patients. Adventitial fibroblasts isolated from pulmonary arteries of chronic hypoxia-induced PH models and PAH patients display a pro-inflammatory and hyper-proliferative phenotype characterized by the expression of myofibroblast markers and cytokine production. Interestingly, the depletion of circulating monocytes reduces adventitial remodeling in hypoxia-induced PH, suggesting that fibrotic remodeling may, at least in part, rely on the recruitment of circulating monocytic mesenchymal precursors [65,66,67].

- Notch pathway

The Notch signaling pathway is implicated in cell proliferation, differentiation, apoptosis, and survival, involving pulmonary vascular remodeling [68]. It consists of four receptors (NOTCH 1-4) and five ligands (JAG-1,2 and DLL-1,3,4) [69]. Recent studies have shown that the Notch signaling pathway is involved in the pathogenesis of both SSc and PAH. Activation of the pathway occurred in the lesional skin of SSc patients and the fibroblasts, splenocytes, skin, and lungs of diseased mice [70,71,72]. JAG-1 is overexpressed in hypertrophic scars and the skin of SSc, promoting collagen release and fibroblast activation [68]. In addition, healthy fibroblasts stimulated by recombinant human JAG-1-Fc chimera led to SSc-like phenotypes with fibroblast differentiation and higher collagen release [70]. Knockdown of JAG-1 results in antiangiogenic activity and the inhibition of keloid fibroblast migration and proliferation [68].

The overexpression of NOTCH 1 and 3 was observed in patients with PH and in both a monocrotaline (MCT)-induced PH rat model and a hypoxia-induced PH mouse model [73,74,75]. JAG-1 promotes PASMC proliferation through NOTCH 3 signaling activation [69]. The γ-secretase inhibitor dibenzazepine interferes with NOTCH 3 signaling, leading to the decreased migration and proliferation of pulmonary artery endothelial cells (PAECs) and the improvement of PAP in mouse PAH models [69,75]. Furthermore, anti-NOTCH3 antibody treatment was able to reverse PAH in both mouse and rat PAH models [69].

NOTCH 2 plays a critical role in the homeostatic processes of the pulmonary endothelium. The expression of NOTCH 2 was attenuated in PAECs when exposed to hypoxia, ET-1, and TGF-β. Additionally, NOTCH 2 levels were reduced in the lungs of PAH patients [76]. Some γ-secretase inhibitors may be beneficial in the therapeutic intervention of PH by blocking Notch pathway activation. AMG2008827 decreased the systolic pressure of the right ventricle and improved RVH in hypoxia/SU5416 rats [75]. DAPT (N-S-phenyl-glycine-t-butyl ester) successfully treated PH in rodents [74]. Moreover, DAPT, with strong antifibrotic effects, diminished the proliferation of fibroblasts and the fibrosis of the skin and lungs in HOCl-injected mice [71].

The Notch pathway regulates fibroblast homeostasis, the process of EndMT, angiogenesis, and the maintenance of SMCs in an undifferentiated state [68,71,74,77]. Considering the crucial role of the Notch pathway in both SSc and PAH development, it may be involved in the pathogenesis of SSc-PAH.

- Hypoxia-inducible factor pathway

The hypoxia-inducible transcription factor (HIF), a crucial regulator of cell response to hypoxia, consists of the oxygen-sensitive α subunits (HIF-1α, HIF-2α, HIF-3α) and the oxygen-insensitive β subunits (HIF-1β, HIF-2β, HIF-3β) [78]. HIF signaling is known to be one of the underlying mechanisms of PAH and contributes to the hypoxic pulmonary vascular remodeling [78,79]. Increased pulmonary expression of HIF-1α, HIF-1β, and HIF-2α has been observed in PAH patients [78]. HIF-1α is regarded as an early marker of generalized hypoxia because it is upregulated in newborns with cyanosis and persistent PH [78].

IL-33 is a member of the IL-1 cytokine family and its expression is stimulated by HIF-1α. The overexpression of IL-33 and its receptor ST2 is observed in PAECs of hypoxic PH patients and hypoxic PH murine models. IL-33/HIF-1α signaling may be involved in the vascular remodeling of hypoxic PH by regulating its downstream factor, the vascular endothelial growth factor (VEGF) [80]. CD146, which mediates cell proliferation and differentiation, is significantly increased in PASMCs of hypoxia-induced PH mouse models and MCT-induced PH rat models. Its levels also correlate with disease severity. The disruption of the CD146-HIF-1α axis alleviates vascular remodeling in chronic hypoxic mice and attenuates PH [81].

HIF-2α also plays an important role in hypoxia-induced PAH [79]. In pulmonary endothelial HIF-2α deletion mice, the typical increase in right ventricular systolic pressure (RVSP) after chronic hypoxic exposure was absent. Endothelial HIF-2α deletion prevented hypoxia remodeling and hypoxia PH in mice [79,82]. Hypoxia is implicated in the pathogenesis of SSc by inducing oxidative stress and the imbalance between oxidants and antioxidants. Nine hypoxia-associated hub genes were identified in the study by He et al., regulating lipolysis, oxidative stress, and the pentose phosphate pathway [83]. HIF-1α levels were significantly elevated in SSc patients [84]. HIF-1α/VEGF signaling regulates hypoxia-induced EndMT on the microvascular remodeling of SSc skin [85]. In a cohort study by Takagi et al., the single-nucleotide polymorphism of the *HIF-1α* gene (AA genotype at rs12434438) was markedly higher in SSc-PAH patients and associated with PAH severity [86].

#### 2.2.3. In Situ Thrombosis

In PAH, in situ thrombosis may be partially attributed to the persistent rise in local hemodynamic stress, elevated ET-1 levels, inflammatory mediators, and certain growth factors, along with a loss of NO and vasoprotective prostacyclin [87,88]. Notably, advanced glycosylation end products and the tumor necrosis factor (TNF)-α have been shown to promote coagulation by downregulating the endothelial anticoagulant cofactor thrombomodulin and inducing the pro-coagulant tissue factor [88,89].

#### 2.2.4. Extracellular Matrix Remodeling

The ECM of a pulmonary artery typically contains collagens, elastins, fibronectin, laminins, proteoglycans, and tenascin C. The imbalance between proteolytic enzymes, such as metalloproteinases, MMPs, lysyl oxidases, serine elastases, and their endogenous inhibitors, tissue inhibitors of metalloproteinase (TIMPs), leads to collagen cross-linking, collagen deposition, and the breakdown of elastin in the ECM of pulmonary arteries. This imbalance also promotes inflammation, angiogenesis, and fibrosis in connective tissues. Expansion of the ECM across all layers of the pulmonary vascular wall leads to fibrosis, resulting in loss of compliance and stiffening of the pulmonary arteries in PAH [90,91].

The altered and increased expression of TIMPs and proteolytic enzymes has been observed in pulmonary arteries from PAH patients and PH animal models compared to controls [91,92,93,94]. Elevated serum TIMP-4 levels were found in SSc patients with pulmonary artery systolic pressure ≥ 40 mmHg in echocardiography [90], and higher serum TIMP-1 levels were associated with increased mortality in IPAH patients [95]. The ECM remodeling of pulmonary arteries in PAH is driven by various mechanisms, including hypoxia, inflammation, *BMPR2* mutation, and increased flow [96].

#### 2.2.5. Right Ventricular Dysfunction

The RV adaptation to pressure overload in PAH plays a crucial role in determining the functional status and prognosis of patients. RVH in PAH is induced by chronic pressure overload and serves as a compensatory response to preserve cardiac output while reducing wall stress. However, in some cases, maladaptive cardiac remodeling may result in RV dilation. A study showed that adult PAH patients with RV failure who were admitted for inotropic therapy had a significantly higher acute mortality rate—up to 41%—compared to those admitted with left ventricular failure [97]. A small left ventricle and RV dilation are independent predictors of a higher mortality rate [98].

Altered glucose metabolism in the right ventricle and the autonomic activation and downregulation of β1 receptors, along with RV ischemia and fibrosis, contribute to the progression of maladaptive RVH and RV failure [99,100]. Patients with congenital heart disease-related PAH typically exhibit adaptive RVH [101], while those with CTD-PAH often experience maladaptive RV remodeling, which leads to more severe RV dysfunction and a higher incidence of RV failure [102]. Maladaptive RVH is also associated with a higher risk of clinical deterioration compared to adaptive RVH in IPAH patients [103].

Moreover, SSc-PAH patients are more likely to develop RV dysfunction [104]. Although whether interstitial fibrosis in the right ventricle differs in SSc-PAH and IPAH is controversial, more inflammatory cells in the right ventricle and impaired sarcomere function were found in SSc-PAH patients [105,106].

### 2.3. Genetics

More studies about whether genetic factors contribute to CTD-PAH have been reported recently. Some heritable genes are important and associated with the pathogenesis of PAH. The most studied gene is *BMPR2*, a member of the TGF-β superfamily. *BMPR2* mutations are identified in 50–80% of FPAH and 10–40% of sporadic PAH [18,107]. Mutations in *BMPR2*, *Endoglin (ENG)*, and Activin A receptor-like type 1 (*ACVRL1*, also known as *ALK-1*) are observed in childhood PAH [108]. However, Morse et al. reported that heterogeneous germline mutations of *BMPR2* were not found in SSc-PAH patients [109].

The *ENG* gene is located at 9q34.1 and is important in maintaining vascular integrity. Elevated serum levels of Eng were found in SSc-PAH patients [37]. A 6-base insertion in intron 7 of the *ENG* gene (6bINS) (5′-TCCCCC-3′) was identified to be associated with microvascular abnormalities. However, the study showed that SSc-PAH patients had a lower frequency of the 6bINS allele of the *ENG* gene compared to HCs. This result indicated that the 6bINS allele is not associated with SSc-PAH [108].

Furthermore, genome-wide association studies and exome sequencing have implicated multiple genes, primarily major histocompatibility complex (MHC) variants and immune pathway-related genes, in the pathogenesis of SSc [110]. However, most of these studies have lacked sufficient numbers of patients with PAH to determine whether genetic variants linked to overall disease risk also contribute specifically to PAH or failed to investigate this phenotype independently. In contrast to heritable PAH, no distinct Mendelian gene associations have been identified for SSc-PAH, and studies on SSc-PAH have not detected mutations commonly reported in hereditary PAH or IPAH.

However, certain genetic factors may still contribute to increased disease susceptibility. For instance, a rare functional variant in *TLR2* Pro631His (rs5743704) was found to be associated with PAH in SSc patients [111]. In addition, *NKX2-5* promoter polymorphisms were genetically associated with PH in scleroderma patients, and *NKX2-5* was identified as a key regulator of SMC phenotypic modulation during pathological vascular remodeling [112]. Furthermore, the potassium voltage-gated channel shaker-related subfamily member 5 (*KCNA5*) gene was involved in vascular tone regulation, and the *KCNA5* rs10744676 variant was associated with SSc-PAH in the Caucasian population [113,114]. These findings suggest that genetic factors may contribute to the development of PH in CTD patients.

### 2.4. Potential Serum Biomarkers

RHC is the gold standard for the diagnosis of SSc-PAH; however, it is an invasive procedure that is sometimes unavailable in non-referral hospitals. Therefore, there is a need to develop non-invasive methods for the early detection and disease monitoring of SSc-PAH. Below are several potential serum biomarkers for SSc-PAH.

- N-terminal pro-brain natriuretic peptide (NT-proBNP)

NT-proBNP, a biomarker indicating neurohormonal activation, is used to assess heart function and monitor HF and has been validated for risk assessment in PAH. In a study by Mathai et al., serum levels of NT-proBNP were markedly increased in SSc-PAH patients compared to those with IPAH, despite similar hemodynamic conditions. Additionally, serum NT-proBNP levels were found to correlate with survival outcomes in patients with SSc-PAH [27,115].

- Adipsin

Adipokines, such as adipsin, leptin, adiponectin, and visfatin, play a role in modulating vascular fibrogenesis and immune activation and are implicated in the pathogenesis of SSc. Elevated levels of adipsin have been linked to PAH in SSc patients, with this association being stronger than that of serum brain natriuretic peptide (BNP) levels. Additionally, increased expression of the adipsin gene was observed in SSc-PAH patients. These findings suggest that adipsin may serve as an emerging biomarker for SSc-PAH and highlight its potential role in the pathogenic link to adipocyte dysfunction [116].

- Lysyl oxidase (LOX)

LOX is an extracellular enzyme that catalyzes the crosslinking of collagen and elastin. LOX expression has been observed in the vascular lesions of IPAH patients. Vadasz et al. reported higher serum LOX levels in SSc patients compared to HCs, as well as in those with very-early-stage SSc. In addition, LOX levels were elevated in patients with an estimated systolic PAP greater than 40 mmHg. A negative correlation was also found between the diffusing capacity of the lung for carbon monoxide (DLCO) and LOX levels. In SSc-PAH patients, the proliferating endothelium of remodeled pulmonary vessels exhibited strong LOX staining. The study suggests that LOX plays a crucial role in SSc-PAH and may serve as a potential biomarker [117].

- Endothelial microparticles (EMPs)

Microparticles (MPs) are small vesicles released by cells in response to activation, injury, or apoptosis, carrying various cellular components. They play a crucial role in intercellular communication, influencing inflammation, coagulation, immune response, and tissue repair. EMPs modulate cellular signaling and contribute to vascular disease development. The concentration of CD144^+^ EMP was higher in SSc-PAH patients compared to those with SSc-nonPAH and HCs. MPs isolated from SSc patients induce a stronger inflammatory response in ECs than those from HCs. EMPs may serve as a novel biomarker for SSc-PAH [118].

- Asymmetrical dimethylarginine (ADMA)

ADMA is an endogenous L-arginine analog that inhibits NOS. Elevated serum levels of ADMA have been observed in SSc-PAH patients compared to those with SSc-nonPAH and HCs. ADMA levels negatively correlated with 6 min walking distance in SSc-PAH patients. These findings suggest that ADMA plays a role in the development of SSc-PAH and may be a potential serum biomarker [22].

More potential serum biomarkers for SSc-PAH are summarized in Table 1.

## 3. Non-SSc CTD-PAH

Since the pathogenesis of non-SSc CTD-PAH closely resembles that of PAH in general, and only a few studies focus specifically on non-SSc CTD-PAH, here, we review the unique findings and observations associated with PAH in various non-SSc CTDs based on the existing literature.

### 3.1. Systemic Lupus Erythematous-Associated PAH (SLE-PAH)

Pathological findings of SLE-PAH are rarely described. Dorfmuller et al. reported the presence of typical vascular lesions, including concentric intimal fibrosis, eccentric intimal fibrosis, laminar concentric intimal fibrosis, loose intimal fibrosis, and in situ thrombosis in lung samples from SLE-PAH patients [16].

In addition to the previously mentioned pathogenesis, several other distinct mechanisms have been proposed in SLE-PAH. A study demonstrated increased brachial–ankle pulse wave velocity, reduced carotid artery strain, and a correlation between anti-cardiolipin immunoglobulin G (IgG) and carotid deformation, indicating that arterial stiffness contributes to the pathogenesis of SLE-PAH [130]. Another study proposed that chronic anemic hypoxia could contribute to elevated PAP in SLE patients, with IL-6 playing a key role in this process [131]. Furthermore, it was demonstrated that impaired BMPR2 signaling and proinflammatory factors collectively contribute to the development of PAH in SLE [125]. The pharmacologic inhibition of macrophage migration inhibitory factor, a precursor inflammatory cytokine, may provide an effective approach to alleviate SLE-PAH [132].

Autoantibodies, including anti-ET_A_R, anti-U1 RNP, and antiphospholipid antibodies (aPL), are linked to SLE-PAH. Anti-ET_A_R autoantibodies are more prevalent in SLE-PAH patients than those without PAH, and they trigger inflammation, cell proliferation, and vasoconstriction, positively correlating with systolic PAP. These autoantibodies promote VSMC proliferation, increase EC monolayer permeability, and stimulate key growth factor expression, suggesting their involvement in SLE-PAH development [133]. Anti-U1 RNP antibodies are also more common in SLE-PAH patients [133,134,135]. A meta-analysis found that aPL increases the risk of SLE-PAH [136]. While the exact mechanisms linking these autoantibodies to SLE-PAH remain unclear, they may help identify high-risk patients.

### 3.2. Primary Sjögren’s Disease-Associated PAH (pSS-PAH)

Using whole-exome sequencing, Li et al. identified 141 pathogenic variant loci in 129 genes from a cohort of 34 pSS-PAH patients. Sanger sequencing confirmation and pathogenicity validation led to the proposal of five candidate variants, which may serve as potential genetic markers for the early detection of pSS-PAH [137]. In a Chinese multicenter cohort, anti-SSB and anti-U1RNP antibodies were recognized as risk factors for developing pSS-PAH. However, the exact role of these antibodies in the pathogenesis of pSS-PAH requires further investigation [138].

### 3.3. Mixed Connective Tissue Disease-Associated PAH (MCTD-PAH)

A clinical and immunoserological study of 179 MCTD patients revealed that MCTD patients with PAH had a lower 5-year survival rate (73%) compared to those without PAH (96%). Anti-endothelial cell antibodies (AECAs) were more prevalent in MCTD-PAH patients, and higher serum levels of thrombomodulin and von Willebrand factor antigen (vWFAg) were observed in these patients, with significant correlations between AECA quantity and both thrombomodulin and vWFAg levels. These findings suggest that AECA and EC activation may play a role in PAH development in MCTD [126].

### 3.4. Rheumatoid Arthritis-Associated PAH (RA-PAH)

In two cohort studies, PAH occurrence in RA patients correlated with RA disease activity and duration [139,140]. The survival rate was similar to that of IPAH patients, even though RA-PAH patients had an older median age at diagnosis and a lower median mPAP at baseline [141]. Yang et al. conducted a Mendelian randomization study examining the link between RA and PAH. Surprisingly, they found that seropositive RA was associated with a lower risk of PAH, while seronegative RA showed no such relationship. The underlying mechanisms are unclear but may involve antibodies in seropositive RA influencing immune processes in PAH or the genetic profile of seropositive RA counteracting PAH development [142].

## 4. Immune Dysregulation in CTD-PAH

Immunity and inflammation are thought to play a key role in PAH, especially in CTD-PAH, such as SSc-PAH and SLE-PAH, and in infectious disease-associated PAH, such as human immunodeficiency virus and schistosomiasis-associated PAH. Dysregulated immune responses and perivascular lymphoid neogenesis and inflammation in the lung are widely recognized as key pathogenic factors in all forms of PAH [143]. Moreover, perivascular and transmural vascular inflammatory infiltrates, especially lymphocytes, were also found in SSc-PAH patients [15].

Multiple studies on the histopathology of IPAH have demonstrated the presence and distribution of both innate and adaptive immune cells within vascular lesions, indicating that perivascular immune cell infiltration contributes to vascular remodeling. Furthermore, evidence suggests that activated vascular cells release increased levels of inflammatory mediators, cytokines, and chemokines, which, in turn, sustain the ongoing recruitment of inflammatory cells—such as macrophages, mast cells, lymphocytes, and dendritic cells (DCs)—to the perivascular space [144,145,146]. Elevated levels of autoantibodies have been implicated in the development of pulmonary vascular lesions and the induction of EC apoptosis. Moreover, antibody-complement deposits, tertiary lymphoid tissues, and circulating autoantibodies are linked to PAH and some autoimmune diseases, such as SSc and SLE [147,148].

### 4.1. Innate Immune Cells

- Macrophages/monocytes

Macrophage accumulation is a key feature of pulmonary arterial remodeling in PH. Studies have shown that the number of infiltrating perivascular macrophages is markedly elevated in both clinical and experimental PAH. These increased macrophages were primarily localized to the adventitial layer of large, medium, and small pulmonary arteries [65,144,145,149].

In PH, adventitial fibroblasts in the pulmonary arteries can recruit, retain, and activate macrophages/monocytes, thereby contributing significantly to chronic inflammatory processes in the vascular remodeling of hypoxic PH [66]. The interaction between macrophages/monocytes and adventitial fibroblasts is supported by evidence showing that fibroblasts from human and experimental PAH activate macrophages via paracrine IL-6-activated STAT3 signaling, driving tissue remodeling, chronic inflammation, and the progression of PH [67]. Moreover, depleting macrophages and blocking macrophage-derived cytokines have been shown to reverse PH in several animal models [150,151].

- Mast cells

Mast cells play a key role in releasing various pro-inflammatory cytokines, proteases, and growth factors. The accumulation of mast cells in the perivascular region has been observed in both clinical PH and experimental PH animal models [145,152,153]. Mast cells are implicated in pulmonary vascular remodeling by releasing substances like MMPs and histamine. Notably, preventing mast cell degranulation with disodium cromoglycate has been shown to reduce the development of hypoxia-induced PH in rats [154].

- Dendritic cells

DCs, the most potent professional antigen-presenting cells, serve as an important link between innate and adaptive immunity. These cells play a crucial role in modulating both immune responses and tolerance.

Perros et al. first reported the presence of immature DCs in the pulmonary arteries of IPAH patients and PH animal models, suggesting their involvement in the immunopathology of PH [155]. In addition, the accumulation of these DCs may contribute to the formation of antibodies against self-antigens. A subsequent study showed that monocyte-derived DCs were impaired in their ability to stimulate T cell proliferation, and there was a significant reduction in DC numbers in the peripheral blood of PAH patients [156].

### 4.2. Adaptive Immune Cells

- T cells

Several studies have reported increased total T cell counts in remodeled pulmonary vasculature [144,145,157]. Further analyses of accumulated T cells showed higher levels of both CD4^+^ and CD8^+^ subsets, while the Foxp3^+^ subset was reduced [145]. The circulating T cells expressing lymphocyte function-associated antigen-1 were elevated in SSc-PAH patients compared to HCs and SSc-nonPAH. In contrast, the proportion of T cells expressing very late antigen-4 or L-selectin was lower in the SSc-PAH group than in HCs and SSc-nonPAH [158].

- CD8^+^ Cytotoxic T cells

Elevated CD8^+^ cytotoxic T cell infiltrates were noted in remodeled pulmonary vasculature, while a decrease in these cells was observed in the peripheral blood of IPAH patients [145,159]. Additionally, distinct CD8^+^ T lymphocyte subsets were observed in IPAH patients, with a notable rise in peripheral cytotoxic effector-memory cells and a decrease in naive CD8^+^ cells [160]. In general, CD8^+^ cytotoxic T cells recognize and bind to MHC class I molecules, leading to the direct killing of target cells. However, the precise role of these cells in the pathogenesis of PAH remains unclear.

- CD4^+^ T cells

In the context of PAH, CD4^+^ T helper (Th) cells are more widely recognized for their involvement in disease pathogenesis compared to CD8^+^ T cells. Pro-inflammatory cytokines, including IL-6, IL-13, IL-17, and TNF, produced by CD4^+^ Th cells, have been shown to contribute to vascular remodeling [161,162,163,164]. IL-7 receptor (IL-7R), a transmembrane protein, is expressed on lymphocytes and monocytes, especially on CD4^+^ and CD8^+^ T cells. IL-7R signaling is responsible for the proliferation, differentiation, and maintenance of lymphocytes. Down expression of IL-7R on CD4^+^ T cells was observed in SSc-PAH patients [165].

- Th1 and Th2

Michael et al. reported that following EC injury, the perivascular infiltration of CD4^+^ T cells contributes to persistent pulmonary artery remodeling in an MCT-induced PH model [146]. Moreover, Th1 cytokine interferon (IFN)-γ and CD4^+^ T cells have been identified as key players in the development of pneumocystis-associated PH in mice [166].

Daley and colleagues found that the Th2 response is responsible for the severity of pulmonary arterial remodeling in antigen-challenged and immunized mice. Notably, the depletion of CD4^+^ T cells, suppression of the antigen-specific Th2 response, or inhibition of IL-13 significantly alleviated pulmonary arterial muscularization [167].

- Th17

Purified CD4^+^ T cells from IPAH patients exhibited elevated IL-17 expression upon activation compared to controls. Furthermore, notable hypomethylation of the IL-17 promoter was observed in the blood DNA of IPAH patients, suggesting Th17 cell immune polarization in these individuals [168]. Th17 cells have also been found in the perivascular region of lungs from chronic hypoxia-induced PH mice, with the Th17/T regulatory (Treg) cells imbalance playing a pivotal role in the progression of chronic hypoxia-induced PH [169,170]. CTD-PAH patients showed an increase in peripheral Th17 cells and a decrease in Treg cells compared to both HCs and CTD patients without PAH. A distinct T cell profile with a higher frequency of Th17 and peripheral Th cells was identified in SSc-PAH patients. Importantly, the Th17/Treg ratio was significantly associated with the prognosis and severity of the disease [171,172].

- Treg

Treg cells are critical immunomodulators in the adaptive immune system and their dysfunction has been observed in idiopathic, heritable, and CTD-PAH [58]. Tamosiuniene et al. showed in animal models that the blockade of VEGF receptor-2 led to the development of significant PH in athymic nude rats, whereas immune-reconstituted rats were protected, indicating the protective role of Treg cells in PH. The dysfunction of Treg cells is thus considered a critical “second hit” in the pathogenesis of PAH [173]. In addition, Treg cell therapy mitigated hypoxia-induced PH in mice, diminished pro-inflammatory cytokines, and increased IL-10 levels in vivo. Treg treatment also notably decreased the proliferation of PASMCs and regulated the cell cycle in vitro [174].

- B cells

Studies have identified that tertiary lymphoid tissues containing B cell follicles and CD20^+^ B cells aggregate in the vessels of IPAH patients [145,175]. Bronchus-associated lymphoid tissues also proliferate in both PH animal models and human PAH, where they play an active role in the production of autoantibodies. Moreover, the passive transfer of autoantibodies from MCT rats to healthy rats has been shown to induce pulmonary vascular remodeling and hypertension [176]. B cells may be involved in SSc-PAH by producing angiogenic mediators and regulating the release of antibodies. Levels of B cell biomarkers can be used to assess disease severity and activity in SSc patients. Higher β2-macroglobulin, soluble CD23 and CD27 levels, and lower serum IgG levels were observed in SSc-PAH patients [177].

- Circulating autoantibodies

A high prevalence of disease-specific antibodies was observed in SSc-PAH patients. For instance, over 80% of SSc-PAH patients tested positive for antinuclear antibodies (ANA), while almost half of these patients showed positive results for anticentromere antibodies (ACA) and aPL [178]. ACA was found to be significantly associated with PAH in SSc patients and was included in the algorithm for detecting SSc-PAH in the DETECT study [10]. Moreover, around 2–14% of SSc patients, 20–40% of SLE patients, and 100% of MCTD patients, by definition, have anti-U1 RNP antibodies [179]. Sobanski et al. found that, among SSc-PAH groups, patients with anti-U1 RNP antibodies were younger at diagnosis and had better mean predicted DLCO; also, a larger proportion of them were in WHO functional classes I and II compared to those without anti-U1 RNP antibodies. A trend toward better survival was also identified in anti-U1 RNP antibody-positive patients, although without statistical significance [180].

More circulating autoantibodies associated with disease development, progression, and prognosis for SSc-PAH/CTD-PAH are listed in Table 2.

- Cytokines and chemokines

Both the immune cells and the vascular cellular components can generate specific cytokines and chemokines, promoting pulmonary vasculopathy [54]. Compared to SSc-nonPAH, SSc-PAH patients had higher IL-6, IL-13, IL-22, IL-32, TGF-β2, and TNF-α levels and lower IL-4 and TGF-β1 levels [36,37,158,192,193,194,195,196]. IL-32 plays a key role in regulating EC activities and is positively correlated with mPAP in SSc patients [195].

IL-18 and its decoy receptor, the IL-18 binding protein (IL-18BP), regulate the activation of IL-18 signaling, which relates to the pathological processes of SSc. Serum IL-18BPa levels were not only elevated in SSc patients but also positively correlated with mPAP, serum erythrocyte sedimentation rate, and C-reactive protein levels. The inhibition of IL-18 signaling may be associated with systemic inflammation in SSc and related PAH [197]. Furthermore, SSc patients at high risk of PAH exhibited cytokine profiles similar to those of SSc-PAH patients. This may suggest that cytokines play a key role in PAH progression and continuation [198].

Increased plasma levels of type I, II, and III IFN were noted in SSc-PAH patients, and, as expected, IFN-stimulated products, interferon γ inducible protein (IP10), and ET-1 were also elevated in SSc-PAH patients. In this group, IP10 was found to be positively correlated with mPAP, PVR, and serum BNP levels [36,37].

Growth differentiation factor (GDF)-15, involved in cell growth and differentiation, is a member of the TGF-β superfamily. Plasma GDF-15 levels were elevated in SSc-PAH patients compared to SSc-nonPAH. A positive correlation between GDF-15 and NT-proBNP was identified. Higher GDF-15 levels were associated with reduced survival in SSc-PAH patients [199].

CD40 and its ligand (CD40L) are members of the TNF receptor superfamily. CD40L is expressed by activated CD4 T cells and binds to CD40 on the B cell surface, mediating B cell activation. CD40L can also be released in a soluble form (sCD40L), which is biologically active and elevated in CTDs [200,201]. Serum sCD40L levels were higher in SSc-PAH patients than in SSc-nonPAH and correlated with estimated PAP [202].

C-C motif ligand (CCL) 20 is an inflammatory chemokine involved in fibrosis and angiogenesis. It can attract effector and memory T cells, B cells, and immature DCs. Serum CCL20 levels showed a positive correlation with mPAP in SSc patients [203]. Chemokine receptor CCR7 and its ligands, CCL19 and CCL21, were found to be involved in PAH development. CCL21 was higher in SSc-PAH patients than in HCs and SSc-nonPAH patients. CCL21 also had predictive value for PAH in SSc patients [204,205].

CXCL16, a proangiogenic chemokine, binds to CXCR6 and promotes angiogenesis. Like other proangiogenic chemokines, such as CXCL1, CXCL8, and CCL2, CXCL16 was upregulated in SSc patients. In addition, CXCL16 was significantly increased in SSc-PAH patients compared to SSc-nonPAH [206]. On the other hand, antiangiogenic chemokines, CXCL4 and CXCL10, were highly expressed in SSc patients. Higher serum CXCL4 levels were also identified in SSc-PAH patients [204,206,207].

More cytokines and chemokines involved in the pathogenesis of SSc-PAH/CTD-PAH are summarized in Table 3.

## 5. CTD-PAH Animal Models

Animal models have been extensively used to help decipher the molecular pathogenesis underlying CTD-PAH and develop new treatments. However, due to the complexity of the disease, no single animal model can reproduce the full clinical spectrum of the disease. In the following section, we describe the setup details, characteristics, and advantages of currently available CTD-PAH animal models (Table 4).

### 5.1. R-SU Rat

Toll-like receptors (TLRs)7/8 are intracellular pattern recognition receptors that selectively recognize single-stranded RNA sequences. Human TLR7 in plasmacytoid dendritic cells (pDCs) is an important trigger for type I IFN signaling. Dysregulated TLR7-mediated IFN production by pDCs drives autoreactive B cell expansion and reshapes Th cell polarization, establishing a hallmark of autoimmunity [208]. Repeated topical application of the TLR7/8 agonist resiquimod (R848) induces SLE-like autoimmune syndrome in mice, leading to increased cytokine levels, autoantibody production, and multi-organ damage [209].

SU5416 is widely known to induce EC apoptosis, a transient but crucial pathogenic event that induces pulmonary vascular remodeling. Combined with other insults, such as chronic hypoxia and T cell depletion, it has been used to generate experimental PH in vivo [210].

The R-SU rat is a novel CTD-PAH animal model established by a combination of R848-induced autoimmunity and SU5416-induced pulmonary endothelial injury. The R-SU rat exhibited severe PAH phenotypes, including significantly elevated mPAP and RVH and typical obliterative vascular remodeling with remarkable perivascular inflammation in the lungs. In addition, this model developed lupus-like syndrome with the upregulation of type I IFN, an increased Th17/Treg ratio, and circulating autoantibodies, resulting in multiple organ involvements, such as splenomegaly and glomerulonephritis [211].

The advantages of this model include being the only rat CTD-PAH model in which RHC is possible, being a drug-induced approach with only a 5-week preparation period, and including immune profiles, such as autoantibodies and peripheral blood mononuclear cells, that closely mimic those observed in SLE-PAH.

### 5.2. Pristane/Hypoxia Mice

Pristane, a mineral oil, is a potential environmental factor in human SLE and is used in SLE mouse models. The PriHx model, induced by pristane administration and 4-week hypoxic exposure, led to increased RVSP, pulmonary vascular remodeling, and RVH, indicating a more severe PH phenotype than that with hypoxia alone. Immune cell accumulation, including macrophages and CD4^+^ T cells, was observed in PriHx mice, along with increased lung fibrosis, reflecting features of both PH groups 1 and 3. These findings suggest that the PriHx model better replicates CTD-PAH than conventional hypoxia-induced PH models [212].

### 5.3. Fra-2 Transgenic Mice

The Fra-2 (Fos-related antigen-2) transgenic (Tg) mouse model exhibits both vasculopathy and fibrosis in the skin and internal organs [213]. Overexpression of the Fra-2 protein is observed in the lungs and skin of SSc patients. Backcrossed onto a pure C57BL/6 background, Fra-2 tg mice develop severe pulmonary vascular remodeling and interstitial lung disease (ILD) resembling SSc-PAH. Histological features include intimal thickening with concentric laminar lesions, medial hypertrophy, perivascular inflammation, and adventitial fibrosis, though pulmonary occlusive venopathy is absent. Furthermore, interstitial inflammation and fibrosis similar to nonspecific interstitial pneumonia, the most common type of SSc-associated ILD (SSc-ILD), are observed. These findings suggest that the Fra-2 Tg model effectively mimics key aspects of SSc-PAH and SSc-ILD, making it a valuable tool for studying disease mechanisms and potential therapeutic targets [214].

### 5.4. Fli1/Klf5 Mice

Friend leukemia integration 1 (Fli1), an Ets transcription factor, represses type I collagen gene expression and mediates a non-canonical TGF-β pathway [215]. In SSc, the epigenetic downregulation of Fli1 in dermal fibroblasts may contribute to fibrosis by partially mimicking TGF-β signaling [216]. However, *Fli1^+/−^* mice do not develop dermal fibrosis, indicating that additional factors are involved. Krüppel-like factor 5 (KLF5), an SP/KLF transcription factor, is a potential contributor, as its expression is reduced in SSc skin, and *Klf5* haploinsufficiency alters fibrotic responses in the heart and kidney [217,218].

Mice carrying a double heterozygous deficiency of *Fli1* and *Klf5* spontaneously develop fibrosis and vasculopathy of the skin and lung, B cell activation, and autoantibody production, closely resembling SSc pathology. While the histologic lesions resemble PH, specific characteristics like RVH or RV pressure measurements have not been documented in these animals [219].

### 5.5. TNF Transgenic Mice (TNF-Tg Mice)

The TNF-Tg 3467 mouse line, which carries a single-copy insertion of the human *TNF-α* transgene, was initially recognized as a model for inflammatory-erosive arthritis [220]. More recently, its cardiopulmonary phenotype has been thoroughly assessed. In addition to exhibiting pulmonary vasculitis, female TNF-Tg mice develop severe, progressive obliterative and fibrotic pulmonary vascular lesions that closely resemble those observed in CTD-PAH [221]. The pathology is characterized by EC loss and vascular smooth muscle proliferation. TNF-Tg mice also exhibit progressively increasing RV pressures that closely mimic human disease, along with severe and progressive right heart pathology. Transcriptome analyses further reveal a significant overlap in gene expression profiles between TNF-Tg mice and CTD-PAH patients. Collectively, these findings establish the TNF-Tg mouse as a robust and reliable animal model for CTD-PAH [222].

**Table 4:** Overview and comparison of CTD-PAH animal models.

Animal Model	R-SU Rat	PriHx Mice	Fra-2 Tg Mice	Fli1/Klf5 Mice	TNF Tg Mice
**Stimuli**	VEGFR antagonist + TLR7/8 agonist	Pristane + chronic hypoxia	Fra-2 transgenic	Combined heterozygosity for Fli1 and Klf5	TNF transgenic
**Setup time**	5 weeks	4 weeks	16 weeks	16 weeks	12 weeks
**Elevated mPAP/RVSP**	Severe	Mild	n/a	n/a	Severe
**RVH** **(RV/LV + S)**	Severe	Moderate	Moderate	n/a	Severe
**Main pulmonary vascular histological findings**	Medial hypertrophy Concentric cellular laminar neointimal lesions Stalk-like complex lesions Occlusive lesions	Medial hypertrophy	Medial hypertrophy Concentric laminar intimal thickening Adventitial thickening	Obliteration of precapillary arterioles Pulmonary veno-occlusive disease-like appearance (interseptal venules)	Severe obliterative and fibrotic pulmonary vascular lesions with occlusion Reduced distal pulmonary vasculature
**Inflammatory cells in the lung**	Abundant perivascular macrophages and mast cells Large BALTs in the lung	Myeloid hemosiderin-laden macrophage aggregation around vessels Increased effector memory follicular helper T cells	Perivascular inflammatory infiltrates (prominently T cell component)	Increased B cell accumulation and collagen deposition in the lung	Significant cellular interstitial infiltrate similar to NSIP pattern
**PBMC**	Treg decrease, Th17 increase, NK decrease, TIMP-1 increase	n/a	n/a	n/a	n/a
**Features other than PH**	Pulmonary interstitial inflammation Glomerulonephritis Massive splenomegaly Positive ANA, anti-dsDNA, AECA	Exacerbated lung fibrosis	Interstitial pulmonary fibrosis Skin fibrosis and decreased dermal vessel density	Interstitial pneumonia with pulmonary fibrosis Dermal fibrosis Positive ANA	Inflammatory arthritis
**Advantages**	Autoimmunity-based Irreversible phenotypes Progressive right ventricular dysfunction in cMRI	Pulmonary vasculopathy, interstitial inflammation, and fibrosis model	Pulmonary vasculopathy, interstitial inflammation and, fibrosis model Irreversible phenotypes	Pulmonary vasculopathy, interstitial inflammation, and fibrosis model Irreversible phenotypes	Inflammatory-erosive arthritis model with PAH Severe and irreversible PAH Right ventricular dysfunction
**PH** **classification**	Group 1, CTD (SLE)-PAH	Group 1 + 3, CTD-PH	Group 1 + 3, SSc-PH	Group 1 + 3, SSc-PH	Group 1, CTD-PAH
**Reference**	[211]	[212]	[214]	[219]	[222]

Abbreviations: AECA, anti-endothelial cell antibody; ANA, anti-nuclear antibody; BALTs, bronchus-associated lymphoid tissues; cMRI, cardiac magnetic resonance imaging; CTD, connective tissue disease; dsDNA, double-stranded DNA; Fli1, friend leukemia integration 1; Fra-2, Fos-related antigen-2; Klf5, Krüppel-like factor 5; LV, left ventricle; mPAP, mean pulmonary arterial pressure; n/a, not applicable; NK, natural killer cells; NSIP, non-specific interstitial pneumonia; PAH, pulmonary arterial hypertension; PBMC, peripheral blood mononuclear cells; PH, pulmonary hypertension; PriHx, pristane/hypoxia; R-SU, resiquimod (R848)-SU5416; RV, right ventricle; RVH, right ventricular hypertrophy; RVSP, right ventricular systolic pressure; S, septum; SLE, systemic lupus erythematosus; SSc, systemic sclerosis; Tg, transgenic; Th17, T helper 17 cells; TIMP-1, tissue inhibitors of metalloproteinase-1; TLR7/8, toll-like receptor 7 and 8; TNF, tumor necrosis factor; Treg, T regulatory cells; VEGFR, vascular endothelial growth factor receptor.

## 6. Conclusions

**Figure 1:**
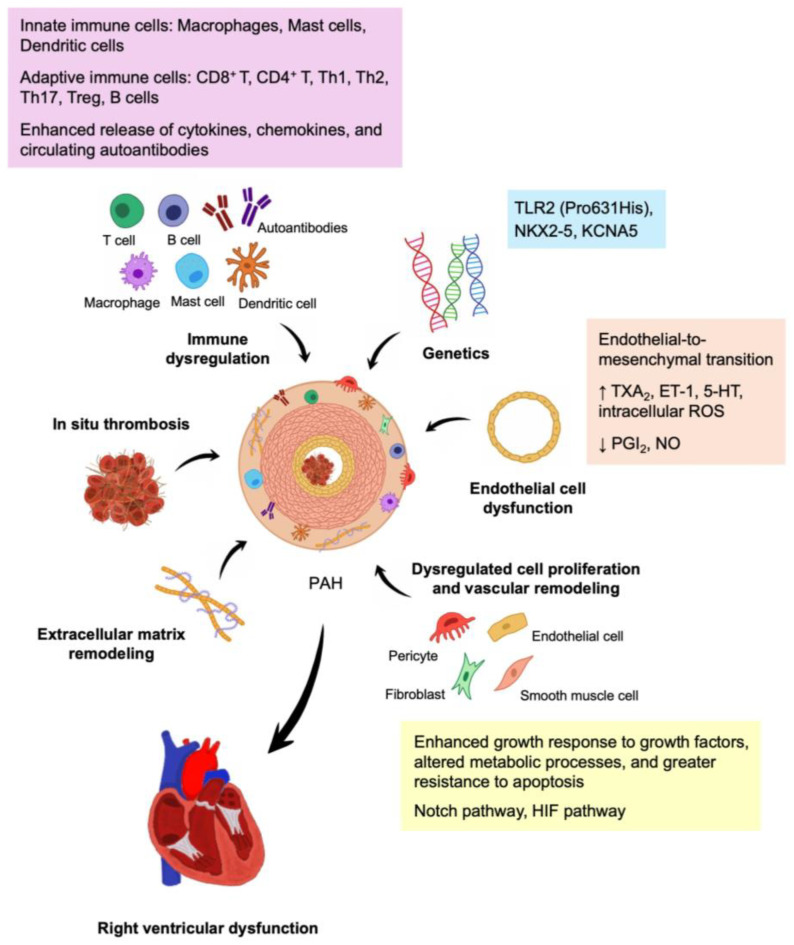
Molecular pathogenesis of CTD-PAH. The key mechanisms involved in CTD-PAH include genetic aberrations, endothelial cell dysfunction, dysregulated cell proliferation and vascular remodeling, extracellular matrix remodeling, in situ thrombosis, immune dysregulation, and right ventricular dysfunction. Abbreviations: 5-HT, 5-Hydroxytryptamine; ET-1, endothelin 1; HIF, hypoxia-inducible factor; NO, nitric oxide; PGI_2_, prostacyclin; ROS, reactive oxygen species; Th, T helper; Treg, T regulatory; TXA_2_, thromboxane A_2_; ↑, increased expression; ↓, decreased expression.

This review provided an overview and summary of the latest molecular mechanisms involved in the pathogenesis of CTD-PAH. Key mechanisms including EC dysfunction, dysregulated cell proliferation and vascular remodeling, in situ thrombosis, ECM remodeling, RV dysfunction, genetic aberrations, and immune dysregulation were identified. Additionally, we explored the role of immune dysregulation in CTD-PAH, highlighting important autoantibodies, cytokines, chemokines, and potential serum biomarkers. A schematic summary of the molecular pathogenesis of CTD-PAH is depicted in Figure 1.

We also emphasized the importance of relevant animal models in advancing our understanding of CTD-PAH and fostering the development of novel therapeutic strategies. With the advancement in diagnostic technologies and molecular medicine, precise gene-level therapies for CTD-PAH, allowing early, personalized treatment, reducing patient suffering, and offering significant benefits, may be anticipated in the near future.

## Figures and Tables

**Table 1:** Potential serum biomarkers for CTD-PAH.

**1.** **SSc-PAH**			
**Serum Levels**	**Biomarkers**		**Reference**
Higher in SSc-PAH patients than in SSc-nonPAH patients	ADMA, CD144^+^ EMP, endostatin, PlGF, soluble Mer, sFlt-1, fatty acyl esters of hydroxy fatty acid, 17β-estradiol, prostaglandin F2α, nervonic acid, a novel eicosanoid		[22,118,119,120,121,122]
Lower in SSc-PAH patients than in SSc-nonPAH patients	miR-26, miR-let-7d		[123]
**Biomarkers**	**Function and Clinical Meanings**		**Reference**
ADMA	Negatively correlated with 6MWD in SSc-PAH patients		[22]
Adipsin	Modulated vascular fibrogenesis and immune activation Involved in SSc pathogenesis Higher levels were associated with PAH in SSc patients		[116]
EMP	Modulated cellular signaling and was involved in the development of vascular disease		[118]
Endostatin	Higher levels were associated with increased mortality in SSc patients		[119]
NT-proBNP	Correlated with survival prediction in SSc-PAH patients		[27,115]
LOX	Serum levels were higher in SSc patients Negatively correlated with DLCO in SSc patients Strong LOX staining in proliferating endothelium of remodeled pulmonary vessels in SSc-PAH patients		[117]
PlGF	Inversely correlated with DLCO in SSc patients		[120,124]
sFlt-1	Positively correlated with right ventricular systolic pressure but negatively correlated with DLCO in SSc patients		[120]
**2.** **Non-SSc CTD-PAH**			
**Serum Levels**	**Biomarkers**		**Reference**
Higher in SLE-PAH patients	Lipopolysaccharide		[125]
Higher in MCTD-PAH patients	Thrombomodulin, von Willebrand factor antigen		[126]
**3.** **CTD-PAH**			
**Serum Levels**	**Biomarkers**	**Function and Clinical Meanings**	**Reference**
Higher in CTD-PAH patients	PTX3	Regulated angiogenesis and cell proliferation A more accurate biomarker for screening PAH in CTD patients than BNP	[127]
	Osteopontin	Associated with inflammation, fibrogenesis, and vascular remodeling process	[128]
	Endostatin, PlGF	Angiogenic and angiostatic factors Inversely correlated with 6MWD	[129]

Abbreviations: 6MWD, 6 min walking distance; ADMA, asymmetrical dimethylarginine; BNP, brain natriuretic peptide; CTD, connective tissue disease; CTD-PAH, connective tissue disease-associated pulmonary arterial hypertension; DLCO, diffusing capacity of the lung for carbon monoxide; EMP, endothelial microparticles; LOX, lysyl oxidase; MCTD-PAH, mixed connective tissue disease-associated pulmonary arterial hypertension; NT-proBNP, N-terminal pro-brain natriuretic peptide; PAH, pulmonary arterial hypertension; PlGF, placental growth factor; PTX3, human pentraxin 3; sFlt-1, soluble vascular endothelial growth factor receptor 1; SLE-PAH, systemic lupus erythematous-associated pulmonary arterial hypertension; SSc, systemic sclerosis; SSc-PAH, systemic sclerosis-associated pulmonary arterial hypertension. Note: Biomarkers in the CTD-PAH section could not be classified as SSc-PAH or non-SSc CTD-PAH because either the types of CTDs were not specified or all CTDs were analyzed together in the studies.

**Table 2:** Circulating autoantibodies for CTD-PAH.

**1.** **SSc-PAH**			
**Antibodies**	**Prevalence and Serum Levels**	**Function and Clinical Meanings**	**Reference**
ANA	Positive in more than 80% of SSc-PAH patients	Hallmarks of CTDs	[178]
Anticentromere antibodies	Positive in almost half of SSc-PAH patients	One of the parameters in the DETECT study’s algorithm for detecting SSc-PAH	[10,178]
aPL	Positive in almost half of SSc-PAH patients	The potential role in thrombosis and other vascular manifestations in SSc patients	[178,181]
Anti-U1 RNP antibodies	Positive in 2–14% of SSc patients	SSc-PAH patients with anti-U1 RNP antibodies were younger at the time of PAH diagnosis and showed better predicted DLCO and WHO functional class	[179,180]
Stimulatory anti-PDGFR autoantibodies	High prevalence of anti-PDGFRα autoantibodies in SSc patients	Converted normal fibroblasts into SSc-like cells in vitro and induced fibrosis in vivo Contributed to migratory activity, the expression of type I collagen genes, and a higher growth rate in human PASMCs in vitro	[61,182,183,184]
Anti-fibroblast antibodies	Positive in 30% of SSc-PAH patients	Activated collagen synthesis by enhancing the expression of ICAM-1	[185,186]
S1PR autoantibodies	Higher prevalence of S1PR2 and S1PR3 autoantibodies in SSc-PAH patients	Mediated cell proliferation, migration, activation, fibrosis, and vasoconstriction	[187,188,189]
Anti-AT_1_R antibodies Anti-ET_A_R antibodies	Higher serum levels in SSc-PAH and CTD-PAH patients than in other forms of PH	Predictive value for PAH development in SSc patients and prognosis in SSc-PAH patients	[32]
**2.** **Non-SSc CTD-PAH**			
**SLE-PAH**			
**Antibodies**	**Prevalence and Serum Levels**	**Function and Clinical Meanings**	**Reference**
Anti-U1 RNP antibodies	Higher prevalence in SLE-PAH patients	Indicated the presence of vasculopathy	[133,134,135]
aPL	Higher prevalence in SLE-PAH patients Higher serum IgG aCL levels in SLE-PAH patients	Associated with the risk of developing PAH in SLE patients Correlated with carotid artery deformation	[130,136]
Anti-ET_A_R antibodies	Higher prevalence in SLE-PAH patients	Titers positively correlated with systolic PAP in SLE-PAH patients Promoted cell proliferation, impaired the endothelial barrier, and stimulated the expression of PAH-associated markers	[133]
**pSS-PAH**			
**Antibodies**	**Prevalence and Serum Levels**	**Function and Clinical Meanings**	**Reference**
Anti-U1 RNP antibodies Anti-SSB antibodies	Higher prevalence in pSS-PAH patients	Associated with the risk of developing PAH in primary Sjögren’s disease patients	[138]
**MCTD-PAH**			
**Antibodies**	**Prevalence and Serum Levels**		**Reference**
AECA IgM aCL	Higher prevalence and serum levels in MCTD-PAH patients		[126]
**3.** **CTD-PAH**			
**Antibodies**	**Prevalence and Serum Levels**	**Function and Clinical Meanings**	**Reference**
AECA	Positive in 63% of CTD-PAH patients Higher serum levels in CTD-PAH patients	Led to upregulation of cytokine secretion and adhesion molecule expression Purified AECA IgG stimulated the secretion and expression of RANTES and ICAM-1 in cultured human PAECs	[190,191]

Abbreviations: aCL, anti-cardiolipin antibodies; AECA, anti-endothelial cell antibodies; ANA, antinuclear antibodies; aPL, antiphospholipid antibodies; AT_1_R, angiotensin type-1 receptor; CTD, connective tissue disease; CTD-PAH, connective tissue disease-associated pulmonary arterial hypertension; DLCO, diffusing capacity of the lung for carbon monoxide; ET_A_R, endothelin receptor type A; ICAM-1, intercellular adhesion molecule 1; MCTD-PAH, mixed connective tissue disease-associated pulmonary arterial hypertension; PAEC, pulmonary arterial endothelial cell; PAH, pulmonary arterial hypertension; PAP, pulmonary arterial pressure; PASMC, pulmonary artery smooth muscle cell; PDGFR, platelet-derived growth factor receptor; PH, pulmonary hypertension; pSS-PAH, primary Sjögren’s disease-associated pulmonary arterial hypertension; RANTES, regulated upon activation normal T-cell expressed and secreted; S1PR, sphingosine-1-phosphate receptor; SLE, systemic lupus erythematous; SLE-PAH, systemic lupus erythematous-associated pulmonary arterial hypertension; SSc, systemic sclerosis; SSc-PAH, systemic sclerosis-associated pulmonary arterial hypertension; WHO, World Health Organization. Note: Antibodies in the CTD-PAH section could not be classified as SSc-PAH or non-SSc CTD-PAH because either the types of CTDs were not specified or all CTDs were analyzed together in the studies.

**Table 3:** Cytokines and chemokines for CTD-PAH.

**1.** **SSc-PAH**			
**Serum Levels**	**Cytokines and Chemokines**		**Reference**
Higher in SSc-PAH patients than in SSc-nonPAH patients	BDNF, CCL21, CXCL16, ET-1, EGF, GDF-15, IL-1β, IL-6, IL-13, IL-22, IL-32, IFN-β, IP10, Leptin, PAI-1, Resistin, sCD40L, TGF-α, TGF-β2, TNF-α, VEGF-D		[36,37,192,193,194,195,198,199,202,204,205,206]
Lower in SSc-PAH patients than in SSc-nonPAH patients	IL-4, TGF-β1		[192]
**Cytokines and Chemokines**	**Function and Clinical Meanings**		**Reference**
CCL 20	Attracted effector/memory T cells, B cells, and immature dendritic cells Mediated fibrosis and angiogenesis Positively correlated with mean PAP in SSc patients		[203]
CCL 21	Predictive value for the development of PAH in SSc patients		[204,205]
CXCL 4	Higher serum levels in SSc patients and correlated with PAH		[207]
GDF-15	Involved in cell growth and differentiation Positively correlated with NT-proBNP, and higher levels were associated with reduced survival in SSc patients		[199]
IL-13	Stimulated the expression of MRC1, which served as a marker of alternative activation of macrophages, regulated the immune response, and correlated with PAP in SSc patients		[193]
IL-17A	SSc patients with detected IL-17A had a higher prevalence of PAH		[196]
IL-18BPa	Related to the pathological processes of SSc Higher serum levels in SSc patients and correlated with mean PAP and serum ESR/CRP levels		[197]
IL-32	Regulated endothelial cell activities and positively correlated with mean PAP in SSc patients		[195]
IP10	Positively correlated with mean PAP, PVR, and serum BNP levels in SSc-PAH patients		[36]
**2.** **Non-SSc CTD-PAH**			
**Serum Levels**	**Cytokines and Chemokines**	**Function and Clinical Meanings**	**Reference**
Higher in SLE-PAH patients	MIF	Mediated cell proliferation and survival	[132]
Higher in MCTD-PAH patients	IL-6	Indicated active acute inflammation	[126]
**3.** **CTD-PAH**			
**Serum Levels**	**Cytokines and Chemokines**	**Function and Clinical Meanings**	**Reference**
Higher in CTD-PAH patients	IL-17	The Th17/Treg axis mediated vascular remodeling and CTD-PAH development, severity, and prognosis Increased IL-17-positive inflammatory cells in the pulmonary artery walls of PAH model rats	[62,171]

Abbreviations: BDNF, brain-derived neurotrophic factor; BNP, brain natriuretic peptide; CCL, C-C motif ligand; CRP, C-reactive protein; CTD, connective tissue disease; CTD-PAH, connective tissue disease-associated pulmonary arterial hypertension; EGF, epidermal growth factor; ESR, erythrocyte sedimentation rate; ET-1, endothelin 1; GDF, growth differentiation factor; IFN, interferon; IL, interleukin; IL-18BPa, IL-18 binding protein a; IP10, interferon γ inducible protein; MCTD-PAH, mixed connective tissue disease-associated pulmonary arterial hypertension; MIF, macrophage migration inhibitory factor; NT-proBNP, N-terminal pro-brain natriuretic peptide; PAH, pulmonary arterial hypertension; PAI-1, plasminogen activator inhibitor-1; PAP, pulmonary artery pressure; PVR, pulmonary vascular resistance; sCD40L, soluble CD40 ligand; SLE-PAH, systemic lupus erythematous-associated pulmonary arterial hypertension; SSc, systemic sclerosis; SSc-PAH, systemic sclerosis-associated pulmonary arterial hypertension; TGF, transforming growth factor; Th, T helper; TNF, tumor necrosis factor; Treg, T regulatory; VEGF-D, vascular endothelial growth factor D. Note: Cytokines and chemokines in the CTD-PAH section could not be classified as SSc-PAH or non-SSc CTD-PAH because either the types of CTDs were not specified or all CTDs were analyzed together in the studies.

## Data Availability

Not applicable.
